# Supplementary material for: A surface-stabilized ozonide triggers bromide oxidation at the aqueous solution-vapour interface
Source: Nat Commun. 2017 Sep 26;8:700. doi: 10.1038/s41467-017-00823-x (PMC5615067; doi:10.1038/s41467-017-00823-x)
Supplement: Supplementary file 1 — Supplementary Information [file 41467_2017_823_MOESM1_ESM.pdf]

Description of Supplementary Files

File Name: Supplementary Information
Description: Supplementary Figures, Supplementary Table, Supplementary Notes and Supplementary Reference

File Name: Peer Review File
Description:

Supplementary Note 1. Heterogeneous kinetics

Determining the uptake coefficient, γ

We follow the general method of describing the heterogeneous kinetics in terms of the uptake coefficient, γ, which is the overall loss rate from the gas phase normalized to the gas kinetic collision rate with the surface in a system, following the concepts outlined in the relevant literature[1-3]. As evident from this definition, γ is not representing an elementary chemical process, but lumps together the multitude of processes involved in the transfer of a molecule from the gas phase to the surface, reactions thereon, transfer into the bulk, diffusion and reaction therein, as discussed further below. However, γ can be obtained experimentally from the measured loss rate of the gas phase reactant, ozone in the present case, assuming first-order kinetic conditions, which has been assured for the setup used in this work[4]. The relevant ozone concentration for uptake is the gaseous ozone concentration near the liquid surface, which is not necessarily the same as the average concentration measured after the trough, due to diffusional gradients. However, for the small uptake coefficients as in this study, limitation of gas diffusion can be neglected[4]. We calculated γ_{obs} as:

$$\gamma_{\text{obs}} = \frac{4 \cdot Q}{\omega_{O_3} \cdot SA_{\text{reactor}}} \times ln\left(\frac{O_{3,\text{bypass}}}{O_{3,\text{reactor}}}\right) \qquad (1)$$

$O_{3,bypass}$ is the measured ozone concentration delivered to the reactor. $O_{3,reactor}$ is the ozone concentration after the reaction has taken place. Q is the flow rate of the gas passing through the reactor ($cm^3\ s^{-1}$). SA is the total surface area of the solution (cm^2)[4].

Predicting the total uptake, γ_{calc}

Within the resistor model concept of heterogeneous kinetics, the general expression derived for a combination of reactive processes on the surface and in the bulk condensed phase is given by[3]:

$$\frac{1}{\gamma} = \frac{1}{\alpha_s} + \frac{1}{\Gamma_s + \left(\frac{1}{\Gamma_{sb}} + \frac{1}{\Gamma_b}\right)^{-1}} \qquad (2)$$

Where Γ_b denotes the limiting uptake coefficient contributed by the reaction in the bulk, Γ_s is the limiting uptake coefficient contributed by the reaction on the surface, Γ_{sb} is the limiting uptake coefficient contributed by the surface to bulk transfer of ozone, and α_s is the surface accommodation coefficient. As mentioned in the main text, γ_{calc} was calculated as direct addition of the bulk and surface reaction contribution[3-5], because it is assumed that neither adsorption to the surface, nor transfer from the surface to the bulk aqueous phase is rate limiting, so that under steady state conditions,

$\gamma_{calc} = \Gamma_b + \Gamma_s$ (3)

We calculated Γ_b using the following equation[1,6]:

$$\Gamma_\mathrm{b} = \frac{4 \cdot R \cdot T \cdot H}{\omega_{O_3}} \times \sqrt{D_\mathrm{liq} \cdot k_\mathrm{b}^I}\,, \quad (4)$$

where R is the gas constant (0.08206 L atm K^{-1} mol^{-1}), T is the temperature (K), H is the Henry constant (M atm^{-1}), D_{liq} is the diffusivity of ozone in the liquid phase (cm^2 s^{-1}), ω_{O3} is the velocity of the ozone molecules in the gas phase (cm s^{-1}), and $k_b^I = k_b^{II} \cdot a_{Br-,b}$ is the pseudo-first order reaction rate coefficient, the product of the second order reaction rate coefficient $k_{b,II}$ (L mol^{-1} s^{-1}) and the Br^- activity in the bulk, $a_{Br-,bulk}$ (mol L^{-1}). It is assumed that $a_{Br-,bulk}$ is not significantly depleted during the experiment. Equation 4 is valid as long as the reacto-diffusive length, l_{rd} = sqrt(D_{liq}/k_b^I), is smaller than the thickness of the solution ($d_{solution}$), which is the case for our experiments ($l_{rd} \leq 10$ µm; $d_{solution} < 4$mm).
For spherical particles, the interplay between diffusion and reaction can be taken into account with a size dependent correction factor, where r_p is the radius of the particle:

$$\Gamma_b = \frac{4 \cdot R \cdot T \cdot H}{\omega_{O_3}} \times \sqrt{D_\mathrm{liq} \cdot k_\mathrm{b}^I} \times \left[\coth\left(r_\mathrm{p}/l_\mathrm{rd}\right) - \left(l_\mathrm{rd}/r_\mathrm{p}\right)\right] \quad (4a)$$

We described Γ_s as a reaction of the Langmuir-Hinshelwood type (Γ_{LH}). Using the resistance model, the contribution of a Langmuir-Hinshelwood surface reaction can be described by[3,7]:

$$\Gamma_\mathrm{LH} = \frac{4 \cdot k_\mathrm{s} \cdot K_\mathrm{Lang} \cdot N_\mathrm{max}}{\omega_{O_3} \cdot \left(1 + K_\mathrm{Lang} \cdot [O_3]_\mathrm{g}\right)}, \quad (5)$$

Where k_s is the apparent second order surface reaction rate coefficient of the adsorbed ozone precursor with bromide, K_{Lang} is the Langmuir constant, N_{max} is the maximum surface coverage (corresponding to about 1×10^{12} molecules cm^{-2}, as obtained from the XPS data of this study, lower than for typical small molecules competing for area on the surface in the adsorbed state[3]), ω_{O3} is the mean thermal velocity of ozone.
Equations 4 and 5 contain parameters that depend on several other variables. For example, H, which describes the solubility of ozone in the solution, depends on T, pH, and the composition of the solution. We parameterized some of these dependencies when calculating the Γ_b and $\Gamma_{LH.}$

Parameterizing Γ_b and Γ_{LH}

The uptake coefficient describes combinations of physical and/or chemical processes, which depend on the composition of the solution, temperature, and pH. The following describes the parameterization of some of the relevant variables as availability of data permits.

Estimating Γ_b

To predict the Γ_b using the model presented in equation (4), we accounted for the following dependences:
a) The activity of the solutes in solution;
b) The influence of pH, temperature, and the solution composition on the ozone solubility;
c) The influence of the pH and temperature on the reaction rate coefficient;
d) The effect of the viscosity of the solutions on diffusivity.

a) Activity of Br^-

The reactivity between Br^- and O_3 depends on the activity of the Br^- ions in the solution. The activity is given by

$$a_{Br-} = c_{Br-} \cdot \gamma_{Br-}, \quad (6)$$

Where c is the concentration and γ is the activity coefficient. At ionic strengths larger than 0.005 mol kg^{-1}, ion-ion interactions become important and the activity coefficient deviates from 1. We calculated the activities of solutes in solutions using the thermodynamic model AIOMFAC[8,9].

b) Effect of solution composition on ozone solubility

The solubility of ozone in the solution depends on the temperature, on the pH, and on the composition of the solution. The lower the temperature and the pH, the higher is the solubility of ozone in the solution[10].

Studies have shown that salts like NaBr tend to decrease the solubility of ozone ("salting-out") in solution[11,12]. To account for this salting effect, the Henry's law constant (H; mol L^{-1} atm^{-1}) or the solubility of ozone in the solution was calculated using the Sechenov relationship[12,13]:

$$log\left(\frac{H_{\mathrm{soln}}}{H_o}\right) = KC_{\mathrm{s}} = \sum(h_i + h_{\mathrm{G}})C_i, \quad (7)$$

H_{soln} is the Henry's law constant of ozone in the solution of interest, H_o is the Henry's law constant of ozone in pure water; K is the Sechenov coefficient; C_i is the concentration of ion, i, (in mol/L), h_i is the ion factor (h_{H+}, h_{Na}, and h_{Br-} were obtained from reference[11], h_G is the gas coefficient, which is temperature-dependent for h_{O3})[14].

We described the temperature dependency of the solubility of ozone in water, H_o, with the empirical function from the National Bureau of Standards[15]:

$$H_o = 1.15 \times 10^{-2} \cdot exp\left(2560 \cdot \left(\frac{1}{T} - \frac{1}{298}\right)\right) \quad (8)$$

Activities in molal concentrations obtained from AIOMFAC were converted to molar concentration using a parameterization of Isono's temperature- and molality-dependent density of NaBr solutions[16].

c) Temperature and pH-dependent rate coefficients, k_b^{II}

IUPAC has proposed a temperature-dependent rate coefficient for the reaction of O_3 and Br^- in the bulk[3,17]. However, studies have shown an enhancement of this reaction at low pH[18]. Liu et al. have proposed a pH-dependent k_b^{II} parameterization at three different temperatures for the scheme below (equation 9), providing values for k_1, k_2/k_{-1}, k_3/k_{-1}, but not for the individual rate coefficients, k_2, k_3, or k_{-1}[18].

$$O_3 + Br^- \overset{k_1/k_{-1}}{\longleftrightarrow} [Br \cdot OOO^-]$$

$$[Br \cdot OOO^-] + H^+ \overset{k_2}{\rightarrow} HOBr + O_2$$

$$[Br \cdot OOO^-] + H_2O \overset{k_3}{\rightarrow} HOBr + O_2 + OH^- ...$$

$$k_b^{II} = \frac{k_1\left(\frac{k_2}{k_{-1}}[H^+] + \frac{k_3}{k_{-1}}\right)}{1 + \frac{k_2}{k_{-1}}[H^+] + \frac{k_3}{k_{-1}}} \quad (9)$$

We have re-parameterized the Liu et al.'s rate coefficients from their k_{obs} measured at different pHs and temperatures to obtain temperature-dependent rate equations (Arrhenius expressions) for each k, which we applied to Liu et al.'s equation for k_{obs}. With this parameterization we obtained 53.9 kJ mol^{-1} as the Arrhenius energy for k_1, which agrees with Liu et al.'s (50 ± 10 kJ mol^{-1}).

$k_1 = 2.07\times10^{13}$ exp(-6480/T);

$k_2 = 1.50\times10^{39}$ exp(-25200/T);

$k_3 = 1.62\times10^{36}$ exp(-24100/T);

$k_{-1} = 4.17\times10^{41}$ exp(-26600/T);

d) Diffusion coefficient of ozone

The diffusion coefficient of ozone in the liquid phase (D_{O3l}) was calculated using the Stokes-Einstein relation, for which the diffusivity is dependent on the viscosity of the

solution (equation 10)[4]. The viscosity depends on the composition of the solution and the temperature[16]. We obtained a temperature and composition fit to Isono's viscosity measurements on NaBr solutions, with which we extrapolated to the relevant temperatures.

$$D_{\mathrm{O3l}} = \frac{k_{\mathrm{B}}T}{6\pi\eta(T)r_{O_3}} \quad (10)$$

For NaBr-HCl mixtures (acidified solution), we used the same viscosity as for the NaBr non-acidified solutions. At pH 1, which corresponds to 0.36 wt%, the effect of HCl on the viscosity of a water-HCl mixture is smaller than 0.5% (estimated from the fitting curves by Nishikata et al.)[19].

Estimating Γ_{LH} (Γ_{s})

In equation 5, for estimating the surface reactivity, the two parameters of concern are K_{Lang} and the k_{s}.

To our knowledge, the only available estimations for K_{Lang} in a bromide-ozone system was presented by Oldridge and Abbatt[5] and in the IUPAC evaluation (based on the same data)[3]. As described in the theory results sections (and confirmed by the photoemission experiments) the pre-transition state species [Br•OOO$^-$] is stabilized by water and has a strong preference for the aqueous solution surface. We have adopted the energy difference between Br^-/water cluster ^{1}[Br^-•4H_2O] and the ozonide at the interface, 5.7 kJ/mol, from the energy diagram as the relevant energy for determining the negative temperature dependence of K_{Lang} (which controls the surface coverage of [Br•OOO$^-$]).

Using this energy, we parameterized a temperature-dependent K_{Lang} in an Arrhenius expression with K_{Lang} observed by Oldridge and Abbatt at 0 °C as a fixed reference:

$$K_{\mathrm{Lang}} = 6.0 \times 10^{-13} \cdot exp\left(\frac{5.7\ kJ\ \mathrm{mol}^{-1}}{RT}\right), \quad (10)$$

Where R is the gas constant (8.314 J mol^{-1}K^{-1}).

The removal of the surface species depends on k_{s}, the rate of the formation of BrO^-, which is limited by the energy difference to the transition state assuming a spin crossing. We therefore adopted the energy difference between the surface species and transition state species assuming spin crossing, 76.4kJ mol^{-1}, for parameterizing k_{s}. We have assumed that k_{s} is independent of the bromide concentration. This assumption is based on the stability of the surface species. Due to this stability, we expect that the surface is mostly saturated with respect to [Br•(O_3)]$^-$ and effectively replenished from the excess in the bulk.

$$k_s = 2.1 \times 10^{12} \cdot exp\left(\frac{-76.4k\text{J mol}^{-1}}{RT}\right)$$
(11)

The measured data of γ_{obs} at the two different temperatures and pH values, and at different bromide concentrations, were fitted simultaneously by equations (2) to (4), with the rate coefficients and K_{Lang} as free variables. We note that several of the parameters are not well constrained by our data alone, since the surface reactivity is likely limited by the product of N_{max}, k_s^{II}, K_{Lang}. However, using available information with respect to the bulk solution properties, in combination with evidence from the present and past kinetic experiments, and taking into account the present spectroscopy and theory results, the kinetic model is able to rationalize the data obtained. The strong stability of the [Br•OOO$^-$] complex at the aqueous solution – vapor interface leads to saturating surface coverage as a function of both gas phase ozone concentration and bulk aqueous phase bromide concentration. This is the only way the surface component of the reactivity can become independent of the bromide concentration, as indicated by the experiment.

Supplementary Note 2. Comparison of measured kinetic data with model fits

Supplementary Figure 1 shows the data measured at 0°C and 5°C used together with the data in the main paper for fitting the model. In this case, the O_3 concentration was measured and used to obtain the measured ozone uptake coefficients.

To further assess the performance of the model, we compared it with Oldridge and Abbatt's measurement at 0 °C, and pH 1.97 (Supplementary Figure 2)[5]. The model seems to underestimate these significantly, especially at intermediate ozone concentrations. However, the disagreement remains within less than a factor of two, and the increase of the uptake coefficient still occurs within the same ozone concentration range. Also, we caution that Oldridge and Abbatt measured the production of $Br_{2(g)}$, which may include further steps in the activation reaction, not accounted for in the model, which treats O_3 loss only. Secondary halogen chemistry both in the boat containing the solution and along the surfaces in their flow system downstream of the boat could have enhanced the Br_2 yields.

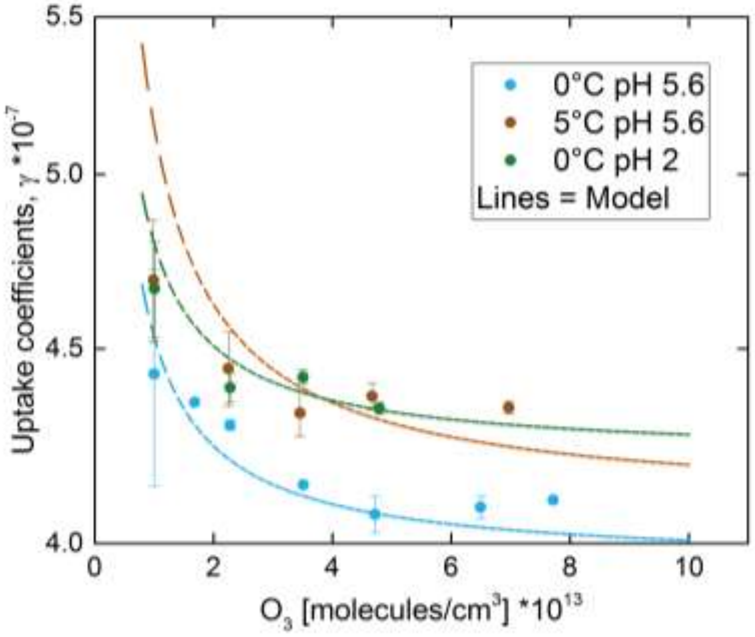

Supplementary Figure 1. Assessment of the model (equation (3)) used to fit the experimental data at different temperatures and pH; all measurements were conducted with 0.125 M NaBr. Error bars represent the standard deviations of measured values.

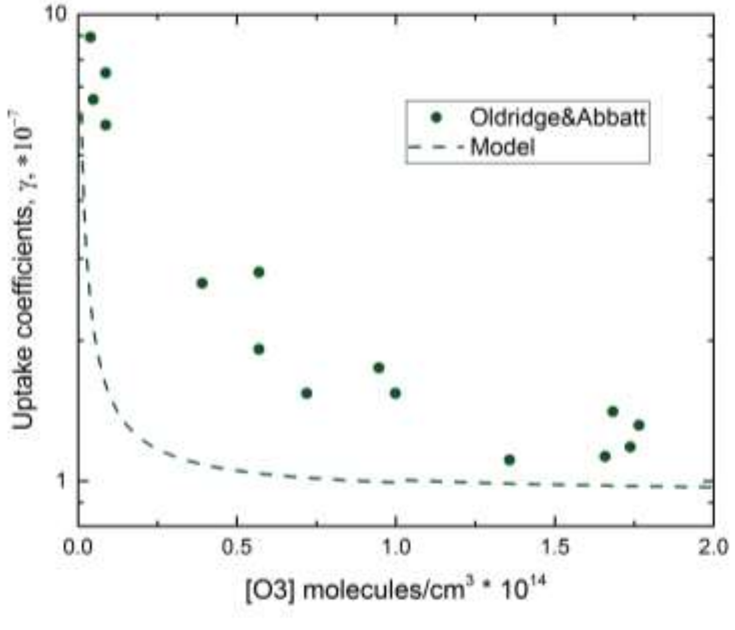

Supplementary Figure 2. Model established by equation (3) compared to the measured data from Oldridge and Abbatt's study[5] at 0°C, pH 1.97, $[KBr^-]$ = 8.6 mM, and [NaCl] = 0.55 M.

Supplementary Note 3. Computational set-up and benchmark

Electronic structure calculations

Electronic structure calculations were performed using the Gaussian09 package[20]
The geometries for the bromide ozone reaction in water were optimized at MP2 level with 6-311++g(df,p) basis set[21]. Harmonic vibrational frequency analysis was used to verify the nature of the optimized structures (minima and saddle points) and to provide the zero-point vibrational energy (ZPE) correction. Supplementary Table 1 reports the Cartesian coordinate for ^{1}[Br●OOO^{-}] optimized geometries, which are shown in the Supplementary Figure 3 with 1, 2, 3 and 4 water molecules.

Supplementary Table 1: Cartesian coordinates in Å for the ^{1}[Br●OOO^{-}] structures, optimized at MP2 level.

Pre complex on singlet – 1 water

O	-2.028302	0.138733	0.726193
O	-1.331408	1.123714	0.087647
O	-2.390859	-0.830960	-0.008698
Br	1.049010	0.055399	-0.201898
O	-1.069093	-0.696775	-2.809647
H	-2.324401	-0.449547	-2.324401
H	-1.701216	-0.694276	-2.084569

Pre complex on singlet – 2 water

O	-1.829179	0.336954	0.567548
O	-1.053554	1.228328	-0.121675
O	-1.842055	-0.849271	0.092971
Br	1.334460	0.409987	0.530750
O	-1.625829	-1.627553	-2.895359
H	-0.940646	-0.961359	-3.046483
H	-1.734378	-1.554131	-1.942769
O	0.199782	0.578217	-2.632505
H	1.002157	0.377054	-2.138821
H	-0.361737	0.864582	-1.896434

Pre complex on singlet – 3 water

O	0.570676	0.890872	-0.034786
O	-0.098421	2.065054	-0.211318
O	0.048454	2.921100	0.720605
Br	-0.838934	-0.330286	1.782212
O	1.074274	1.956665	1.074274
H	0.505113	1.192273	3.232861

H	0.947062	2.446327	2.581750
O	1.406903	-0.758554	1.406903
H	2.285485	-0.003269	0.855335
H	1.628665	-1.033053	1.681871
O	3.732763	0.777858	3.615718
H	3.503776	0.144443	2.924617
H	2.906484	1.274392	3.686174

Pre complex on singlet – 4 water

O	0.114819	2.017727	-1.781213
O	-0.087542	1.323295	-0.629811
O	-0.351804	1.455881	-2.825809
Br	1.616914	-0.614865	-0.752859
O	-1.642114	1.297322	1.790612
H	-1.084111	1.573737	1.056675
H	-2.224534	0.638899	1.378187
O	-0.211715	-1.273567	2.087916
H	-0.458498	-0.355742	2.261192
H	0.427101	-1.185151	1.366838
O	-2.755410	-1.102994	0.694493
H	-2.011572	-1.482342	1.182887
H	-2.461076	-1.182559	-0.227129
O	-1.487014	-1.248374	-1.862975
H	-0.577567	-1.371597	-1.552270
H	-1.410255	-0.365879	-2.242040

In order to improve the energetic, single point energy calculations were performed at CCSD(T)/6-311++g(df,p) level, on top of the previously optimized structures at the MP2 level[22]. Possible multi-reference character for this reaction was already addressed in our previous publication showing negligible effects on the structures and reaction profile[23]. Previous results by Gladich et al. at the CCSD(T)/6-311++g(df,p)//CCSD/6-311++g(df,p) levels in the gas phase reaction were used as benchmark[23]. Supplementary Figure 4 shows the gas phase reaction profile at CCSD(T)//MP2 level. Comparison with Ref. 23 shows that the key features and order of the reaction intermediates are preserved and also the relative energies are reasonable close to the benchmark ones.

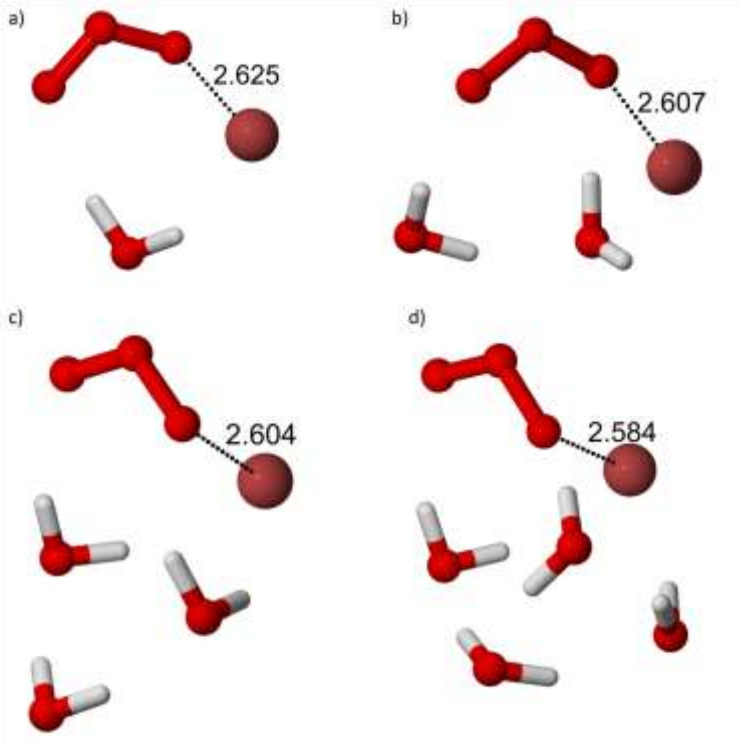

Supplementry Figure 3. $^1[Br\bullet OOO^-]$ optimized geometries at MP2/6-311++g(df,p) level with one, two, three and four water molecules in panels a), b), c) and d), respectively. The figure also show the distance between the bromine atom and the closest $^1[Br\bullet OOO-]$ oxygen.

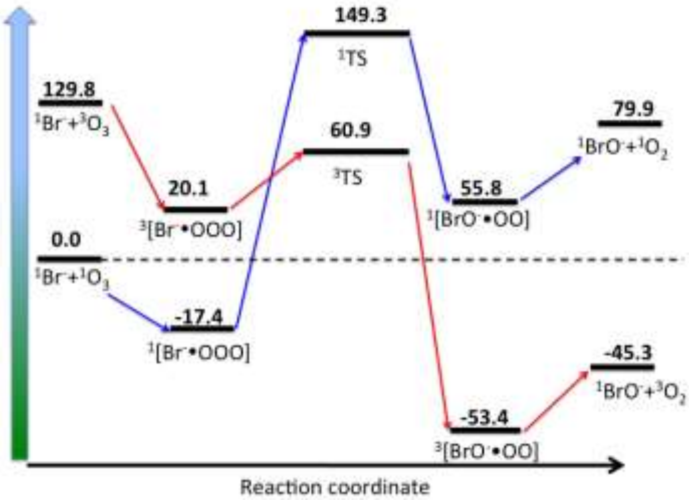

Supplementary Figure 4. Reaction profile for the gas-phase reaction of bromide with ozone along the singlet (blue) and triplet (red) surface obtained at CCSD(T)//MP2 level using 6-311++g(df,p) basis set. All energies are relative to the singlet reactants, corrected by adding the Zero Point Energy (ZPE) and expressed in kJ/mol.

Supplementary Figure 5 shows the reaction profile in 1, 2, 3, and 4 water clusters, indicating that the addition of water stabilizes the pre-complex compared the reaction level, while the barrier on the single and triplet transition states remains high.

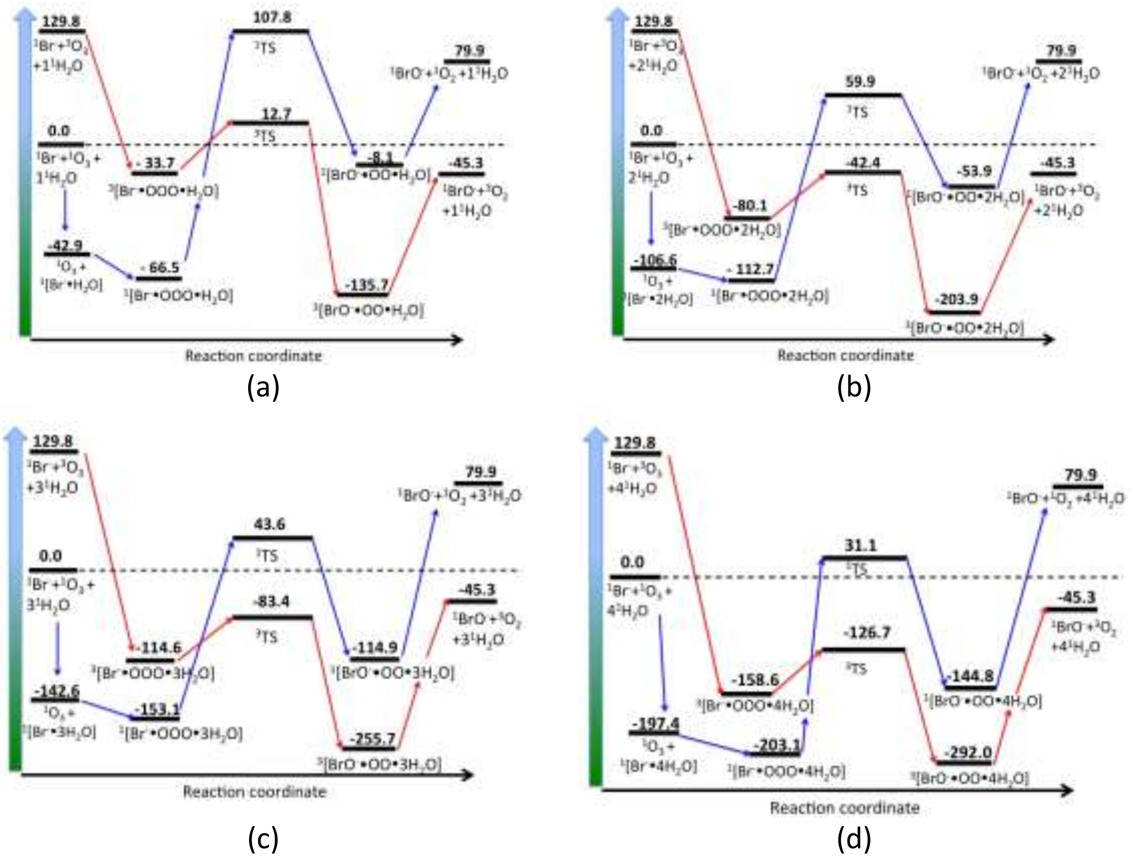

Supplementary Figure 5. Reaction profile for the bromide-ozone reaction along the singlet (blue) and triplet (red) surface obtained at CCSD(T)/MP2 level with 6-311++g(df,p) basis set. Panels **a, b, c** and **d** show the energetic profiles for the different intermediates with one, two, three and four water molecules, respectively. All energies are relative to the singlet reactants and are reported in kJ/mol, including Zero Point Energy (ZPE) correction.

First Principle Molecular Dynamics Simulations

First Principle molecular dynamics simulations based on density functional theory were performed using the CP2K code to study the stability and dynamics of the $^1[Br\bullet OOO^-]$ on the surface of water[24]. The $^1[Br\bullet OOO^-]$ structure obtained at electronic structure level (see above) was placed on the top of an equilibrated water slab of 216 water

molecules on a simulation cell of 20 Å × 20 Å × 60 Å dimension. An optimization of the initial condition was then preformed in order to avoid artificially large forces. Next, a molecular dynamics simulation was carried out in a constant volume and temperature ensemble, using BLYP functional[25,26] with Grimme dispersion correction[27]. Temperature was kept at 300 K by using a Nose-Hoover thermostat. While the valence electrons were explicitly taken into account, Goedecker-Teter-Hutter pseudopotentials were used to model core electrons[28]. The Gaussian-type orbital basis set DZVP was used in combination with plane wave representation, with an energy cutoff at 400 Ry. Similar set-up has been already adopted in literature for studies of liquid water interfaces with and without surfactants[29,30].

Core Electron Binding Energy Calculations

Core electron binding excitation (CEBE) energies were calculated on top of MP2/6-311++g(df,p) geometries. Additionally, it is assumed that the core-hole state geometries are identical to the corresponding ground states. The effect of relaxation was described by the ΔMP2 approach where the electron correlation was described using MP2/aug-cc-pVTZ level of theory[31,32]. The CEBE is calculated as the difference between the ground state energy and the cation core-hole state formed by ejection of Br^- 3d electron. Freezing the molecular orbitals during the SCF procedure prevented the collapse of the core-hole state to the more stable energetic state. All CEBE calculations were performed using GAMESS suite of codes[33].

Supplementary Note 4. Liquid-jet x-ray photoelectron spectroscopy

Technical and experimental details

A gas dosing system, developed by Microliquids GmbH, was connected to the liquid jet assembly. A second nozzle, made of titanium, was screwed concentrically to the quartz nozzle delivering the liquid (see Figure 3a). The outer nozzle has a 500 µm orifice, which is placed 1.0 mm far from the aperture of the quartz nozzle. The gas (either pure oxygen or a mixture of 1% ozone in oxygen, generated in situ by means of a commercial ozone generator (502, Fischer Technology), whose flow was adjusted by means of a variable leak valve connected to the nozzle via a 1/16” PEEK tube, reached the nozzle and was interacting efficiently with the liquid wire in the area prior to injection into the experimental chamber. The efficiency of O_2 to O_3 conversion in the ozone generator was checked in separate experiments by means of the same ozone analyzer as described in the context of the kinetic experiments above. The purity of the gas was checked by means of a quadrupole mass spectrometer installed in the second differential pumping stage of the electron analyzer. During the experiment, we made use of two cold traps (liquid nitrogen) and a 27 $m^3 h^{-1}$ root pump. Before dosing the gas, the pressure in the experimental chamber was $3 \cdot 10^{-2}$ mbar. While dosing the gas, we set the pressure to 0.25 mbar. The advantage of using a gas delivery nozzle, is that we can expect a higher local pressure in the area between the orifice of the quartz nozzle and that of the

titanium one. The diffusion of the gas into the liquid is fast enough that gas –liquid equilibrium over the probe depth of the XPS experiments is established within 1 μs. Because the solubility of ozone is low, the net flux into the liquid is low, and no gradients will limit this flux in the gas phase. The residence time of the liquid filament within the dosing system is approx. 100 μs and between the dosing system and the point at which it hits the X-ray is 100 μs. Therefore, the gas – surface and gas – liquid equilibria follow the changing pressure in the gas phase. A preliminary experiment carried out without the gas dosing system showed a similar evolution of the Br 3d spectrum while dosing ozone (0.1-0.2 mbar background pressure). However, the effect was less pronounced than after employing the gas dosing system, indicating that loss of ozone to the chamber walls has a significant effect on the gas exposure on the liquid. Concerning the diffusion of bromide ions in the solution, the evolution of the surface concentration of an initially free surface by diffusion from the bulk with time is given by equation 12:

$$c_s = 2\left(\frac{D}{\pi}\right)^{1/2} c t^{1/2} \qquad (12)$$

where c is the bulk concentration of the bromide solution ($7.53 \cdot 10^{19}$ molecules cm^{-3}), c_s is the surface concentration of $[Br \bullet OOO^-]$ (estimated as $2 \cdot 10^{12}$ molecules cm^{-2}), and D is the diffusion coefficient ($2.41 \cdot 10^{-10}$ $cm^2 s^{-1}$), evaluated through the Stokes-Einstein equation. The time needed to re-establish the equilibrium concentration is approx. 2 μs, much lower than the time between the injection of the liquid filament and the detection point (100 μs).

The Swiss light source synchrotron operates in top-up mode, thus the photon flux did not change during measurements. It is well known from the literature that X-rays can induce the radiolysis of water, leading to the formation of highly reactive hydroxyl radicals (reference 34 and references therein), which could affect the measurements. Thanks to the high speed of the injected liquid filament, the liquid microjet technique limits the beam damage, and the concentration of radicals does not reach $1 \cdot 10^{-6}$ mol/L under the experimental conditions adopted in this study.

The 0.125 mol L^{-1} solutions of bromide and bromate were prepared by adding sodium bromide (NaBr, ≥99.0%, Sigma-Aldrich) and sodium bromate ($NaBrO_3$, ≥99.5%, Sigma-Aldrich), respectively, to Milli-Q water (Millipore, 18.2 MΩ cm^{-1} at 25 °C). The 0.08 mol L^{-1} hypobromite solution was prepared by disproportionation of Br_2 in a 0.5 mol L^{-1} solution of NaOH[35]. Hypobromite and bromide were generated based on the reaction:

$Br_2 + 2\ OH^- \rightarrow Br^- + BrO^- + H_2O$.

Hypobromite is thermodynamically unstable and disproportionates to bromide and bromate. To slower this reaction, we cooled the solution to 0°C both during the preparation and the experiment. According to our spectra (Figure 3c), bromate was not formed for the whole duration of the experiment.

The photoemission spectra were fitted using Shirley background subtraction and Gaussian line shapes[4]. For Br 3d, the spin-orbit split was fixed at 1.03 eV. The Full width at half maximum (FWHM) of the peaks was constrained for the whole set of processed data (1.05 eV). A single doublet allowed getting a good deconvolution of the Br 3d signals acquired without dosing and while dosing oxygen. In the presence of ozone, a second doublet had to be added to reach the same correlation. The second doublet is made of Gaussian peaks having a spin-orbit split fixed at 1.09 eV and FWHM of 1.10 eV. The best correlation was obtained applying a chemical shift of +0.7 eV to the binding energy of the second doublet.

Evolution of the O 1s spectrum as a function of the excitation energy

The spectra reported in the Supplementary Figure 6a show the O 1s spectra (excited by second order photon light) acquired at increasing excitation energy, which corresponds to increasing kinetic energy of the photoelectrons. The three kinetic energies are the same as those of the Br 3d spectra plotted in Figure 4c (manuscript). The spectra were separated into two components: one at high binding energy (centered at 539.9 eV), associated to the gas phase of water, and another, centered at 537.9 eV, associated to the condensed phase of water. The latter was used to normalize the area of the Br 3d signal of the [Br●OOO$^-$] complex (Figure 4d). Water being the solvent, it can be considered as a reference during the measurements. The Supplementary Figure 6b shows the ratio between the area of the peak of the oxygen gas phase and that of the condensed phase. The ratio decreases as the kinetic energy of the photoelectrons increases. Indeed, an increase of the kinetic energy of the photoelectrons corresponds to an increase of the probed depth. As long as the energy is increased, a larger thickness of the liquid wire is probed, while the contribution of the gas phase remains constant, apart from changes due to changes in photon flux and cross section (the pressure in the experimental chamber was constant during the whole duration of the experiment).

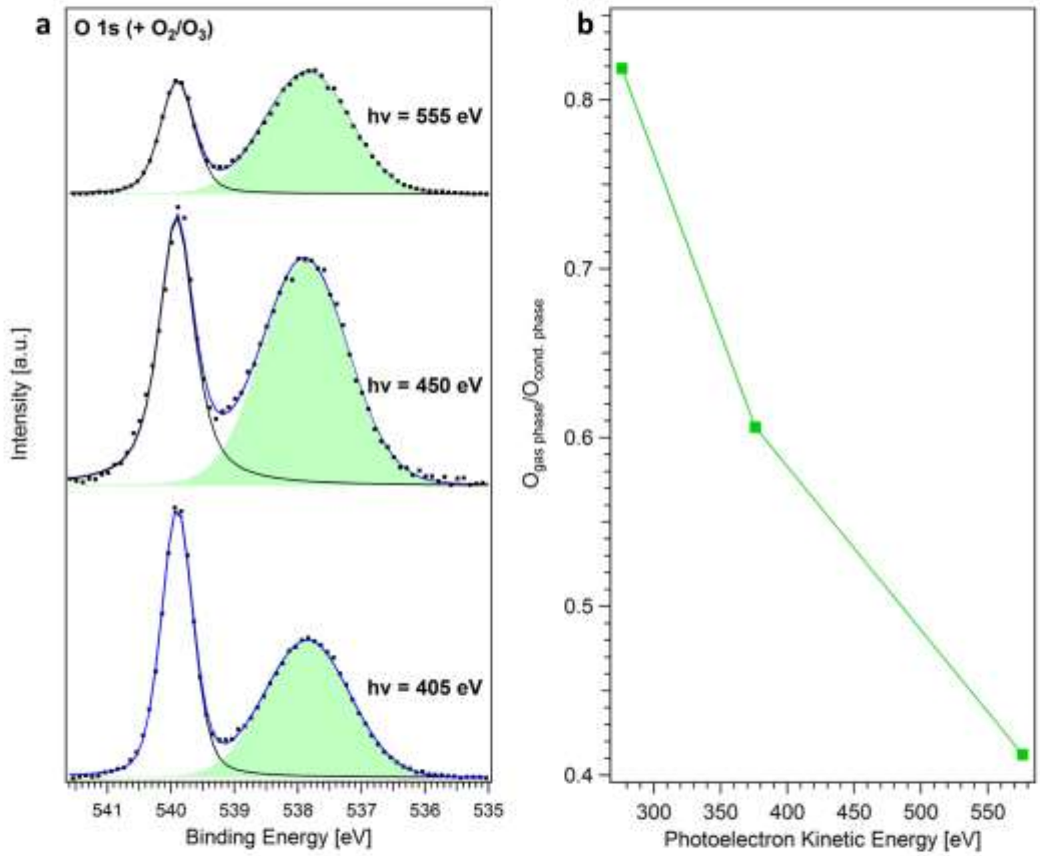

Supplementary Figure 6. Signal of the O 1s acquired at different excitation energies, hv=405, 450, and 555 eV (corresponding to the second order excitation energies hv=810, 900, and 1110 eV), corresponding to the kinetic energies (peak centroid) of 276, 376 and 575eV, respectively.

Evaluation of the [Br●OOO$^-$] surface coverage

Supplementary Figure 7 shows a rough estimate of the surface coverage of [Br●OOO$^-$] complex based on the XPS data acquired at increasing photoelectron kinetic energy (276, 376, and 576 eV). We employed two models to fit the behavior of the ratio between the intensity of the peaks related to the complex (I_{BrOOO^-}) and that of the peaks related to the bulk 0.125 mol L^{-1} solution of Br^- (I_{Br-}) (see Figure 4c). Passing from 276 to 376 eV there is not a significant decrease of the I_{BrOOO}-/I_{Br-} ratio, and this reflects on the quality of the fitting (the experimental point corresponding to 376 eV is out of the confidence interval). A possible reason is that the difference in the excitation energy (100 eV) is not enough to vary considerably the information depth.
The first model (Supplementary Figure 7a and b) does not take into account the attenuation of the photoemission signal from the bulk by the overlayer ([Br●OOO$^-$] complex). The formula adopted is the following:

$$\frac{I_{[\mathrm{Br}\cdot\mathrm{OOO}^-]}}{I_{\mathrm{Br}^-}} = \frac{n([\mathrm{Br}\cdot\mathrm{OOO}^-],\mathrm{surface})}{n(\mathrm{Br}^-,\mathrm{bulk})\cdot MED} \tag{13}$$

where *n([Br·OOO⁻], surface)* is the surface coverage of the [Br●OOO⁻] complex (complexes/cm^2), *n(Br⁻, bulk)* is the ion density of bromide ions in the solution (ions/cm^3), and MED is the mean escape depth of the photoelectrons emitted from the ions (defined as $< MED >_{avg} = 2/\pi \cdot IMFP$, where *IMFP* is the inelastic mean free path of the photoelectrons at each kinetic energy)[36]. We estimated the surface coverage employing two sets of IMFPs. In 2010, Ottosson et al. obtained an experimental curve of the electron attenuation length (EAL) in the 70-900 eV range for water[37]. Recently, Shinotsuka et al. calculated the electron inelastic mean free path for liquid water from its optical energy-loss function[38]. The values are different from each other, thus we calculated the surface coverage using Shinotsuka's IMFPs in Supplementary Figure 7a, and Ottosson's EALs in Supplementary Figure 7b. The surface coverages are 1.9 and 2.9 · 10^{12} complexes cm^{-2}, respectively.

We adopted a second model to fit the experimental data available, which takes into account the attenuation of the signal from the bulk aqueous solution by a thin surface layer of [Br●OOO⁻] complex. The simplified equation, inspired by the literature[39,40], is the following:

$$\frac{I_{[\mathrm{Br}\cdot\mathrm{OOO}^-]}}{I_{\mathrm{Br}^-}} = \frac{\rho_{[\mathrm{Br}\cdot\mathrm{OOO}^-]}}{n(\mathrm{Br}^-,\mathrm{bulk})} \cdot \left[1 - exp^{(-\frac{t}{MED})}\right] \cdot exp^{(\frac{t}{MED})} \tag{14}$$

where $\rho_{[Br\cdot OOO-]}$ is the ion density of the [Br●OOO⁻] complex (complexes cm^{-3}), and *t* is the thickness of the surface layer ([Br●OOO⁻] complex). Supplementary Figure 7 c and d show the results attained using the MED from Shinotsuka and Ottosson, respectively. Three fitting functions, obtained employing three pre-set values of *t* (in the 1.0 to 5.0 Å range) are showed. The best correlation is found using the lower values, i.e. 1.0 and 3.0 Å, proving that the complex does not diffuse toward the bulk. The surface coverages (average value between the three thicknesses, obtained multiplying $\rho_{[Br\cdot OOO-]}$ by *t*) are in good agreement with the results shown in Supplementary Figure 7a and b. The values, 1.7 and 2.6·10^{12} complexes cm^{-2}, decrease by ca. 10% as compared to those obtained with the first model, because of the attenuation.

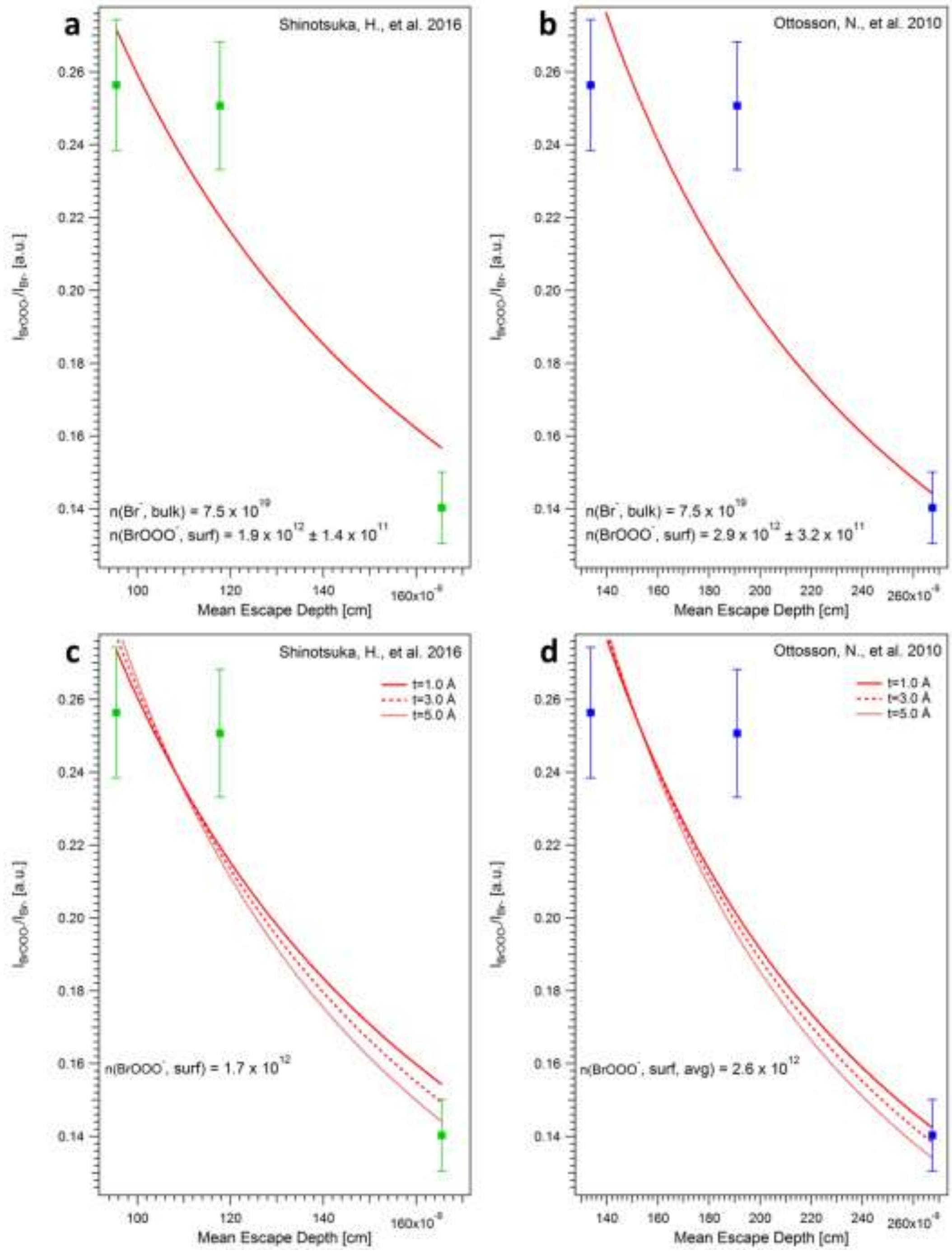

Supplementary Figure 7. Evaluation of the surface coverage of the [Br●OOO-] complex (a)-(b) Simple approximation, without considering the surface attenuation due to the [Br●OOO-] complex; (c)-(d) considering the attenuation of the signal from the bulk (0.125 mol L^{-1} aqueous solution of Br-) by a surface layer containing the [Br●OOO-] complex. The error bars were calculated propagating the errors associated to the peak areas of three different measurements.

Supplementary References

1 Poschl, U., Rudich, Y. & Ammann, M. Kinetic model framework for aerosol and cloud surface chemistry and gas-particle interactions - Part 1: General equations, parameters, and terminology. *Atmos. Chem. Phys.* **7**, 5989-6023 (2007).

2 Kolb, C. E. *et al.* An overview of current issues in the uptake of atmospheric trace gases by aerosols and clouds. *Atmos. Chem. Phys.* **10**, 10561-10605, doi:10.5194/acp-10-10561-2010 (2010).

3 Berkemeier, T. *et al.* Kinetic regimes and limiting cases of gas uptake and heterogeneous reactions in atmospheric aerosols and clouds: a general classification scheme. *Atmos. Chem. Phys.* **13**, 6663-6686, doi:10.5194/acp-13-6663-2013 (2013).

4 Lee, M. T. *et al.* Competition between organics and bromide at the aqueous Solution-Air Interface as seen from ozone uptake kinetics and X-ray photoelectron spectroscopy. *J. Phys. Chem. A* **119**, 4600-4608, doi:10.1021/jp510707s (2015).

5 Oldridge, N. W. & Abbatt, J. P. D. Formation of gas-phase bromine from interaction of ozone with frozen and liquid NaCl/NaBr solutions: Quantitative separation of surficial chemistry from bulk-phase reaction. *J. Phys. Chem. A* **115**, 2590-2598, doi:10.1021/jp200074u (2011).

6 Hanson, D. R., Ravishankara, A. R. & Solomon, S. Heterogeneous Reactions in Sulfuric-Acid Aerosols - a Framework for Model-Calculations. *J. Geophys. Res.* **99**, 3615-3629, doi:Doi 10.1029/93jd02932 (1994).

7 Ammann, M., Poschl, U. & Rudich, Y. Effects of reversible adsorption and Langmuir-Hinshelwood surface reactions on gas uptake by atmospheric particles. *Phys. Chem. Chem. Phys.* **5**, 351-356, doi:10.1039/b208708a (2003).

8 Zuend, A., Marcolli, C., Luo, B. P. & Peter, T. A thermodynamic model of mixed organic-inorganic aerosols to predict activity coefficients. *Atmos. Chem. Phys.* **8**, 4559-4593 (2008).

9 Zuend, A. *et al.* New and extended parameterization of the thermodynamic model AIOMFAC: calculation of activity coefficients for organic-inorganic mixtures containing carboxyl, hydroxyl, carbonyl, ether, ester, alkenyl, alkyl, and aromatic functional groups. *Atmos. Chem. Phys.* **11**, 9155-9206, doi:10.5194/acp-11-9155-2011 (2011).

10 Battino, R., Rettich, T. R. & Tominaga, T. The solubility of oxygen and ozone in liquids. *J. Phys. Chem. Ref. Data* **12**, 163-178 (1983).

11 Weisenberger, S. & Schumpe, A. Estimation of gas solubilities in salt solutions at temperatures from 273 K to 363 K. *AlChE J.* **42**, 298-300, doi:DOI 10.1002/aic.690420130 (1996).

12 Bin, A. K. Ozone solubility in liquids. *Ozone-Sci. Eng.* **28**, 67-75, doi:10.1080/01919510600558635 (2006).

13 Setschenow, J. Z. Uber Die Konstitution Der Salzlösungen auf Grund ihres Verhaltens zu Kohlensäure. *Z. Physik. Chem.* **4**, 117-125 (1889).

14 Rischbieter, E., Stein, H. & Schumpe, A. Ozone solubilities in water and aqueous salt solutions. *J. Chem. Eng. Data* **45**, 338-340, doi:DOI 10.1021/je990263c (2000).
15 Chameides, W. L. Possible Role of No3 in the nighttime chemistry of a cloud. *J. Geophys. Res.* **91**, 5331-5337, doi:DOI 10.1029/JD091iD05p05331 (1986).
16 Tamamushi, R. & Isono, T. A Quasithermodynamic approach to the viscous-flow of aqueous-electrolyte solutions at moderate and high-concentrations. *J. Chem. Soc. Farad.* **80**, 2751-2758, doi:DOI 10.1039/f19848002751 (1984).
17 Haag, W. R., Hoigne, J. & Bader, H. Improved ammonia oxidation by ozone in the presence of bromide ion during water-treatment. *Water Res.* **18**, 1125-1128, doi:Doi 10.1016/0043-1354(84)90227-6 (1984).
18 Liu, Q. *et al.* Kinetics and mechanisms of aqueous ozone reactions with bromide, sulfite, hydrogen sulfite, iodide, and nitrite ions. *Inorg. Chem.* **40**, 4436-4442, doi:10.1021/ic000919j (2001).
19 Nishikata, E., Ishii, T. & Ohta, T. Viscosities of aqueous hydrochloric-acid solutions, and densities and viscosities of aqueous hydroiodic acid-solutions. *J. Chem. Eng. Data* **26**, 254-256, doi:DOI 10.1021/je00025a008 (1981).
20 Frisch, M. J. *et al. Gaussian, Inc. Rev. E01* (2009).
21 Moller, C. & Plesset, M. S. Note on an approximation treatment for many-electron systems. *Phys. Rev.* **46**, 0618-0622, doi:DOI 10.1103/PhysRev.46.618 (1934).
22 Raghavachari, K., Trucks, G. W., Pople, J. A. & Replogle, E. Highly correlated systems: Structure, binding energy and harmonic vibrational frequencies of ozone. *Chem. Phys. Lett.* **158**, 207-212, doi:10.1016/0009-2614(89)87322-1 (1989).
23 Gladich, I. *et al.* Ab Initio Study of the Reaction of Ozone with Bromide Ion. *J. Phys. Chem. A* **119**, 4482-4488, doi:10.1021/jp5101279 (2015).
24 Hutter, J., Iannuzzi, M., Schiffmann, F. & VandeVondele, J. cp2k:atomistic simulations of condensed matter systems. *Wiley Interdisciplinary Reviews: Comp. Molec. Sci.* **4**, 15-25, doi:10.1002/wcms.1159 (2014).
25 Lee, C., Yang, W. & Parr, R. G. Development of the Colle-Salvetti correlation-energy formula into a functional of the electron density. *Phys. Rev. B* **37**, 785-789, doi:10.1103/PhysRevB.37.785 (1988).
26 Becke, A. D. Density-functional exchange-energy approximation with correct asymptotic behavior. *Phys. Rev. A* **38**, 3098-3100, doi:10.1103/PhysRevA.38.3098 (1988).
27 Grimme, S., Antony, J., Ehrlich, S. & Krieg, H. A consistent and accurate ab initio parametrization of density functional dispersion correction (DFT-D) for the 94 elements H-Pu. *J. Chem. Phys.* **132**, 154104, doi:10.1063/1.3382344 (2010).
28 Goedecker, S., Teter, M. & Hutter, J. Separable dual-space Gaussian pseudopotentials. *Phys. Rev. B* **54**, 1703-1710, doi:10.1103/PhysRevB.54.1703 (1996).
29 Baer, M. D. *et al.* Re-examining the properties of the aqueous vapor–liquid interface using dispersion corrected density functional theory. *J. Chem. Phys.* **135**, 124712, doi:10.1063/1.3633239 (2011).

30 Baer, M. D., Tobias, D. J. & Mundy, C. J. Investigation of interfacial and bulk dissociation of HBr, HCl, and HNO3 using density functional theory-based molecular dynamics simulations. *J. Phys. Chem. C* **118**, 29412-29420, doi:10.1021/jp5062896 (2014).
31 Holme, A., Børve, K. J., Sæthre, L. J. & Thomas, T. D. Accuracy of calculated chemical shifts in carbon 1s ionization energies from single-reference ab initio methods and density functional theory. *J. Chem. Th. Comp.* **7**, 4104-4114, doi:10.1021/ct200662e (2011).
32 Shim, J., Klobukowski, M., Barysz, M. & Leszczynski, J. Calibration and applications of the ΔMP2 method for calculating core electron binding energies. *Phys. Chem. Chem. Phys.* **13**, 5703, doi:10.1039/c0cp01591a (2011).
33 Schmidt, M. W. *et al.* General atomic and molecular electronic structure system. *J. Comput. Chem.* **14**, 1347-1363, doi:10.1002/jcc.540141112 (1993).
34 George, G. N. *et al.* X-ray-induced photo-chemistry and X-ray absorption spectroscopy of biological samples. *J. Synchr. Radiat* **19**, 875-886, doi:10.1107/S090904951203943x (2012).
35 Polak, H. L., Fennstra, G. & Slagman, J. Stability of hypobromite solutions. *Talanta* **13**, 715-&, doi:Doi 10.1016/0039-9140(66)80006-1 (1966).
36 Winter, B. & Faubel, M. Photoemission from liquid aqueous solutions. *Chem. Rev.* **106**, 1176-1211, doi:10.1021/cr040381p (2006).
37 Ottosson, N., Faubel, M., Bradforth, S. E., Jungwirth, P. & Winter, B. Photoelectron spectroscopy of liquid water and aqueous solution: Electron effective attenuation lengths and emission-angle anisotropy. *J. Electron. Spectrosc. Relat. Phenom.* **177**, 60-70, doi:10.1016/j.elspec.2009.08.007 (2010).
38 Shinotsuka, H. *et al.* Calculations of electron inelastic mean free paths. XI. Data for liquid water for energies from 50eV to 30keV. *Surf. Interface Anal.*, doi:10.1002/sia.6123 (2016).
39 Fadley, C. S., Brundle, C. R., Baker, A. D. *Electron Spectroscopy: Theory, Techniques and Applications*. Vol. 2 (Academic Press, 1978).
40 Cimino, A., Gazzoli, D. & Valigi, M. XPS quantitative analysis and models of supported oxide catalysts. *J. Electron. Spectrosc. Relat. Phenom.* **104**, 1-29, doi:Doi 10.1016/S0368-2048(98)00300-4 (1999).
